# The outcome of a test-treat package versus routine outpatient care for Ghanaian children with fever: a pragmatic randomized control trial

**DOI:** 10.1186/1475-2875-13-461

**Published:** 2014-11-26

**Authors:** Emmanuel Ameyaw, Samuel B Nguah, Daniel Ansong, Iain Page, Martine Guillerm, Imelda Bates

**Affiliations:** Department of Child Health, Komfo Anokye Teaching Hospital, Kumasi, Ghana; Institute of Inflammation and Repair, The University of Manchester, Manchester, UK; Liverpool School of Tropical Medicine, Liverpool, Upool School of Tropical Medicine, Liverpool, UK

**Keywords:** Children, Fever, Malaria, Infection, Point-of-care test

## Abstract

**Background:**

Over-diagnosis of malaria among African children results in mismanagement of non-malaria infections. Limited laboratory capacity makes it difficult to implement policies that recommend pre-treatment confirmation of infections so a new approach with a package for on-the-spot management of fevers was evaluated.

**Methods:**

Febrile children presenting to outpatient clinic were randomized to receive either a ‘test-treat’ package (history with clinical examination; point-of-care tests; choice of artesunate-amodiaquine, co-amoxiclav and/or paracetamol) or routine outpatient care in a secondary health care facility in Kumasi, Ghana. A diagnosis of malaria, bacterial, viral or mixed malarial and bacterial infections was made using pre-defined criteria. Outcome was resolution of all symptoms including fever on day 7.

**Results:**

The median age of the patients was 37.5 months (IQR: 19 to 66 months), with 56.7% being males. Compared to routine care the test-treat package resulted in less diagnoses of malaria, (37.2% *vs* 46.2%, p = 0.190) and mixed malaria and bacterial infections (14.0% *vs* 53.8%, p < 0.001) but more diagnoses of viral (33.1% *vs* 0.0%, p < 0.001) and bacterial infections only (15.7% *vs* 0.0%, p < 0.001). Less anti-malarials (51.2% *vs* 100.0%, p < 0.001) and antibiotics (29.7% *vs* 48.7%, p < 0.001), were prescribed in the test-treat group on completion of study, more test-treat package patients were clinically well (99.2% *vs* 80.7%, p < 0.001) and febrile (0.8% *vs* 10.1%, p = 0.001) and less were admitted for inpatient care (0.0% *vs* 8.4% p = 0.001) compared to the routine care group.

**Conclusion:**

Test-treat package improves the effectiveness of outpatient diagnosis and treatment of children with fever and reduces inappropriate prescribing of anti-malarials and antibiotics. The package provides clinicians with the option for immediate diagnosis and treatment of non-malaria fevers. The test-treat package now needs to be evaluated in other settings including primary health care facilities.

## Background

The treatment of febrile children makes up approximately two thirds of the paediatric outpatient workload in African hospitals [1]. Malaria should be considered in all febrile children from endemic areas because it is associated with significant morbidity and mortality [2]. Bacterial infections, such as pneumonia, urinary tract infection, tonsillitis and otitis media, are also common causes of childhood fever and may be responsible for more child mortality than malaria, even in malaria endemic areas [3]. When left untreated, these bacterial infections can lead to permanent organ damage and death. However, many of these febrile children will have self-limiting viral infections. They will not benefit from anti-malarial drugs and/or antibiotics and should not be put at unnecessary risk of side effects [3].

Appropriate prescribing or withholding of drugs for infections is not only important for effective treatment and to avoid unnecessary side effects but it is also essential to minimize the risk of organisms developing drug resistance. Although many hospitals have access to microscopy for the diagnosis of malaria [4], fever in children is often treated presumptively as malaria [1, 5]. Consequently, over diagnosis of malaria is common [6] and could have contributed to widespread resistance to anti-malarials, such as chloroquine [7, 8]. Antibiotic resistance is also widespread [9, 10] and it is, therefore, essential that clinicians obtain a definitive diagnosis of fever so that the right drugs can be prescribed.

To be able to rationally prescribe or withhold anti-infective agents clinicians need to have access to diagnostic tests for the most common infections and to use the test results appropriately as guide. Trials have demonstrated the effectiveness of ‘point-of-care’ tests such as malaria Rapid Diagnostic Tests (RDT) and urine dipstick tests for providing specific diagnoses of infections [6, 11] in the African clinic setting [12, 13]. RDTs for *Plasmodium falciparum* malaria provide accurate diagnosis within a few minutes [14–16] and anti-malarial treatment can be safely withheld if the result is negative [3]. Urine dipstick tests have good specificity for infection, and provide a significant improvement on clinical assessment alone in settings where culture is not available [12, 17]. In this study, the feasibility of a simple test-treat package for children with fever presenting to outpatient clinic in a Ghanaian district hospital was assessed to find out whether the package resulted in better clinical outcome than the existing routine care. The test-treat package was based on a structured clinical examination and easily available point-of-care tests and drugs. The package content was compliant with national treatment guidelines so that if it is proved to be effective it has the potential to be scaled up.

## Methods

### Study site

The study was conducted at the outpatient clinic of Suntreso Government Hospital (SGH), a secondary health care facility in Kumasi, Ghana between August and October 2009 during the high malaria transmission season. SGH is a district hospital with 57 inpatient beds. In 2007, 22,322 children were seen and treated at the SGH outpatient clinic and 55.7% were treated as malaria. The hospital laboratory performed blood films for malarial parasites, haemoglobin estimations, automated blood cell counts, urinalysis and stool microscopy, but had no microbiological culture facilities, and clinicians did not have access to RDTS.

### Diagnosis and treatment

All children between 6 months and 12 years of age presenting to the out-patient department with a temperature above 37.5°C or a history of fever were recruited into the study after their caregivers have signed informed consent form. Children requiring admission or referral due to severe illness at presentation as defined by World Health Organization’s (WHO) Integrated Management of Childhood Illness (IMCI) criteria [18] were excluded. Children were randomized to receive either the test-treat package (intervention group) or routine outpatient hospital care (control group). Computer generated 1:1 randomization was done in blocks of 20 patients and used as the basis for the randomization. The results of randomization were placed in sealed envelopes which were opened only when a patient was enrolled into the study. The patients in the test treat package were managed by two clinicians from KATH whereas the patients in the routine care group were managed by the clinicians at SGH. The clinicians in the two groups had the same level of training and experience.

The test trest-package consisted of rapid diagnostic test for malaria, urine dipstick test for urinary tract infection (UTI) and examination of ear, nose and throat, chest and treat with antipyretic with or without anti-malarials and/or antibiotic for any patient presenting with fever or history of fever. This package assumes that urine dipstick test for UTI is comparable to urinalysis, and rapid diagnostic test is comparable to blood film for malaria parasites.

Routine care comprised of outpatient registration, triage of patients by nurses, and care given by attending doctors to patient which consists of history, general examination, followed by either laboratory request or direct treatment based on history and examination by doctors at SGH. The test treat package (intervention) was tested against routine out patient management of fever by doctors at SGH (control). Patients randomized to the test-treat group underwent a structured clinical examination consisting of ear, nose, throat and joint inspection and chest auscultation after their history had been taken. A bed side malaria RDT for all Plasmodium species (Parascreen; Zephyr Biomedicals, India) was performed according to the manufacturer’s specifications. Thick and thin blood films for malaria was done for all participants in the intervention group. All the RDTs were done and interpreted by one of the authors (EA) to eliminate inter-observer bias. A urine sample was obtained from each child in the intervention group, either spontaneously or by catheterization for children under two years old who could not produce urine on demand. These were then immediately tested at the bedside for nitrites and leucocytes using combi-10 urine dipsticks. Samples that tested positive for nitrite and/or leucocytes were transported immediately to the microbiology laboratory of the local Teaching Hospital (KATH) for culture and sensitivity.

Diagnoses made as a result of these clinical and laboratory examinations were based on predefined criteria (Table 1). Patients were thus categorized as having “malaria only”, “bacterial infection only”, “possible viral infection” or “mixed malaria and bacterial infection”. Patients diagnosed as “malaria only” were treated with a combination of artesunate and amodiaquine (Coarsucam®, Sanofi aventis) in accordance with Ghana Health Service guidelines [19]. Those diagnosed as “bacterial infection only” were treated with an amoxicillin-clavulanic acid combination (Amoksiclav®, Sandoz). This antibiotic was selected because of its effective cover for common childhood infections such as respiratory tract infections, urinary tract infections and bacteraemia [20, 21]. Children with diagnosis of “malaria and bacterial infection” received both artesunate and amodiaquine and amoxicillin-clavulanic acid. A patient with history of fever and normal clinical examination, negative malaria RDT and negative urine dipstick results was diagnosed as having a “possible viral illness” and received paracetamol only because such patient did not need antibiotic and/or anti-malarial.

**Table 1:** **Definition and treatment of illnesses in febrile children in intervention group**

Diagnosis	Definition	Treatment
Malaria infection	Positive malaria RDT	Artesunate-amodiaquine for 3 complete days
Bacterial infection	Clinical signs of infection on examination of ears, throat and joints, or chest auscultation (e.g. redness, exudates, inflammation, lower chest wall in-drawing, signs of consolidation) and/or urine dipstick positive for nitrites and/or leucocytes	Amoxicillin-clavulanic acid for 7 days
Possible viral infection	Normal clinical examination, negative malaria RDT and negative urine dipstick	Paracetamol (as required)

Patients in the routine care group received the routine care provided by the clinicians at SGH. These clinicians manage patients based on the recommendation of the Ghana Health Service treatment guidelines [19]. Decisions on clinical examinations, investigations and drug prescriptions were left completely to the discretion of the attending clinician in accordance with their routine management practices. A wide range of antibiotics and anti-malarials were available to the clinicians at SGH. They were not informed about the content of the test-treat package and they did not know that they were involved in the study. This was to prevent them from changing their routine practice of fever management of children (Hawthorne effect). The clinical decisions of the clinicians in the routine group were not interfered by the clinicians in the intervention group. There was also no interchange of patients or clinicians between the intervention and control groups.

Blood and urine samples collected from patients in the intervention group were transported to a laboratory at KATH. The blood film slides were examined independently by two experienced microscopists for the presence of malaria parasites. Discrepant results were reviewed by a third microscopist who determined the final result. The purpose of comparing RDT and microscopy results was to determine the accuracy of the malaria RDT in the study setting. Urine samples were cultured to determine the specific pathogenic organisms and the antimicrobial sensitivity. The management of the children were based on the results of malaria RDT and urine dipstick test as well as clinical examination after history.

### Patient follow up and clinical outcomes

Patients from both groups who received anti-malarial(s) and/or antibiotic(s) were requested to return for review on the second and the seventh day after recruitment. However, all patients, in the intervention group, who received paracetamol only were given appointments for daily follow up as the sensitivity of the RDT used in the study was less than 100%. Sensitivity of the RDT used in this study was 98.0% and specificity is 83.3%. Patients in both groups who failed to attend their follow up appointments were visited at their homes to ensure they were clinically safe. At these reviews all children underwent a clinical assessment and the result documented. The patients were assessed by the same sets of clinicians who had conducted the initial assessment and management in the respective groups.

The primary end point for the study was an asymptomatic and afebrile child as reported by the caregiver/child on day 7. Secondary end points were no symptoms and no fever on day 2. Information was collected about the presence of fever and/or symptoms on days 2 and 7. Patients who had fever at the end of the seventh day were further assessed and managed appropriately. They were followed till recovery. All data were collected on a pre-designed form and entered in a Microsoft Access® 2007 database.

### Statistical analyses

Pre-trial power calculations indicated that 120 patients would be needed in each of the intervention and routine care groups to detect a difference of 15% in the absolute treatment failure rates between the two groups. In this sample size there was an estimation of a two sided type I error of 0.05, a power of 80%, an absolute failure rate of 25% in the routine treatment group and an anticipated non-response/dropout rate of 15% were used. The data were transferred from Microsoft Access 2007 to Stata 10.1 (StataCorp, Texas 77845, USA) from which univariate analysis with point estimates and 95% confidence intervals of baseline characteristics of the children were calculated. Bivariate analysis was performed using the Fischer’s test. Based on blood film analysis, sensitivity, specificity, positive predictive value and negative predictive value for the RDT compared to malaria parasite blood film microscopy (gold standard) in this setting were also calculated.

## Results

### Initial diagnosis and treatment

**Figure 1:**
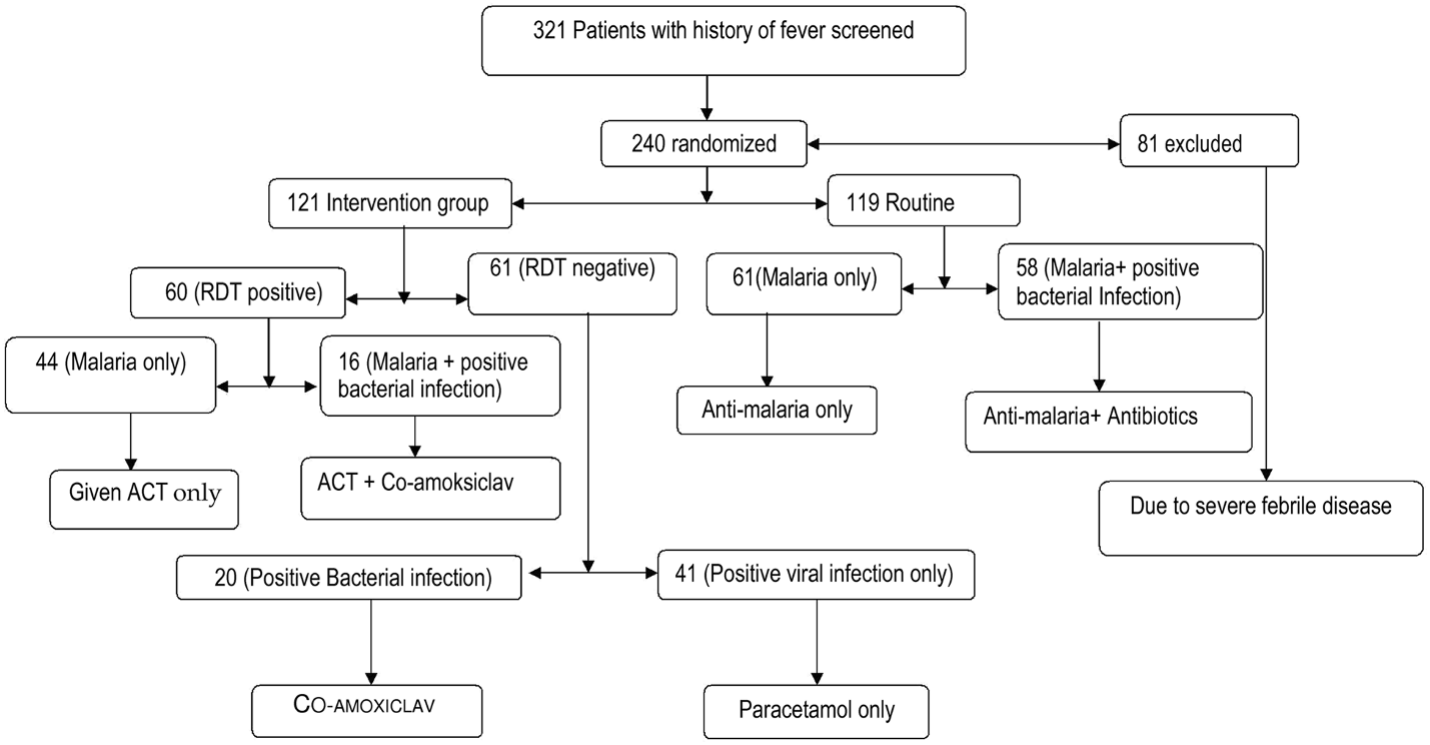
**Trial profile.**

The median age of the patients in the study was 37.5 months (IQR: 19 to 66 months) with 56.7% being males. The median age, age range and sex distribution were similar in both groups. One patient was inadvertently recruited into the intervention group resulting in 121 patients in intervention group and 119 in the routine care group (Figure 1). The caregivers of the patients in both groups had similar educational levels and previous medication histories (Table 2). Overall 175 (72.9%, 95% CI: 66.8 to 78.4%) of the 240 patients had taken medication in the week preceding the study. 160 (66.7%, 95% CI: 60.3 to 72.6%) had taken paracetamol, 15 (6.2%, 95% CI: 3.5% to 10.1%) had amodiaquine, 12 (5.0%, 95% CI: 2.6 to 8.6%) had amoxicillin and 7 (2.9%, 95% CI: 1.2% to 5.9%) had artesunate. Some clinical features differed between the two groups with poor feeding, diarrhoea, cough, inflamed tonsils and rhinorrhoea occurring more frequently in the routine care group. The severity of febrile illness was comparable among the two groups. The mean (and standard) deviations of temperature and respiratory rates in the intervention and control groups at recruitment were 38.5 (0.9); 38.5 (0.9); and 32.0 (10.2); 31.3 (11.1), respectively.

**Table 2:** **Characteristics of children in the intervention and routine care groups at enrolment**

	Study group		
	All patients (N = 240)	Test-Treat (N = 121)	Routine care (N = 119)
**Educational level of carers**			
None	30 (12.5%)	17 (14.0%)	13 (10.9%)
Primary	123 (51.2%)	66 (54.5%)	57 (47.9%)
Secondary School	66 (27.5%)	26 (21.5%)	40 (33.6%)
Tertiary	21 (8.8%)	12 (9.9%)	9 (7.6%)
**Medication taken prior to study**			
Any Drug	175 (72.9%)	86 (71.1%)	89 (74.8%)
Paracetamol	160 (66.7%)	78 (64.5%)	82 (68.9%)
Amodiaquine	15 (6.2%)	6 (5.0%)	9 (7.6%)
Amoxicillin	12 (5.0%)	9 (7.4%)	3 (2.5%)
Artesunate	7 (2.5%)	4 (3.3%)	2 (1.7%)
Co-amoxiclav	0 (0.0%)	0 (0.0%)	0 (0.0%)
Other drugs	47 (19.6%)	24 (19.5%)	23 (19.7%)
**Clinical features at initial assessment**			
Fever	239 (99.6%)	120 (99.2%)	119 (100.0%)
Temperature, *mean (sd)*	38.5 (0.9)	38.5 (0.9)	38.5 (0.9)
Poor feeding	165 (68.7%)	75 (62.0%)	90 (75.6%)
Cough	106 (44.2%)	38 (31.4%)	68 (57.1%)
Respiratory rate, *mean (sd)*	31.4(10.9)	32.0 (10.2)	31.3 (11.1)
Rhinorrhoea	92 (38.3%)	20 (16.5%)	72 (60.5%)
Vomiting	92 (38.3%)	43 (35.5%)	49 (41.2%)
Headache	64 (26.7%)	34 (28.1%)	30 (25.2%)
Diarrhoea	42 (17.5%)	12 (9.9%)	30 (25.3%)
Chills	39 (17.0%)	13 (10.7%)	26 (23.4%)
Inflamed tonsils	14 (5.8%)	13 (10.7%)	1 (0.8%)
Inflamed Eardrum	4 (1.7%)	4 (3.3%)	0 (0.0%)
Sweating	14 (5.8%)	4 (3.3%)	10 (8.4%)
Body pains	10 (4.2%)	4 (3.3%)	6 (5.0%)
Ear pain	6 (2.5%)	3 (2.5%)	3 (2.5%)
Others	12 (5%)	10 (7%)	2 (2%)

At initial assessment, all 121 patients in the intervention group had malaria RDT performed but only one of the 119 patients in the routine care group had a blood film requested for malaria screening. Urine dipstick was positive for nitrites and/or leucocytes in 16 (13.2%, 95% CI: 7.7% to 20.6%) patients in the intervention group. Only one patient in the routine care group had urinalysis performed and it was negative.

There were significant differences among diagnosis between the groups in diagnosis at presentation (Table 3). Similar numbers of patients in both groups, 45 (37.2%) in the intervention group and 55 (46.2%) in the routine care group, were diagnosed as having malaria alone but more children in the intervention group were diagnosed with suspected viral infection (40[33.1%] compared to 0[0%]) or possible bacterial infection only (19[15.7%] compared to 0[0%]). Fewer children in the intervention group than in the routine care group had malaria and bacterial co-infection (17[14.0%] compared to 64[53.8%]). In the routine care group all children were prescribed anti-malarial drug either alone (61[51.3%]) or in combination with an antibiotic (58[48.7%]). Only 51.2% of children in the intervention group received anti-malarials either alone (45[37.2%]) or in combination with an antibiotic (17 [14.0%]). None of the children in the routine group were prescribed either paracetamol alone or an antibiotic alone compared to the intervention group in which 40 (33.1%) received paracetamol alone and 19 (15.7%) received an antibiotic alone.

**Table 3:** **Management and clinical outcomes of children in the intervention and routine care groups**

	Study group			
	All patients (N = 240)	Intervention (N = 121)	Routine care (N = 119)	P-value
**Initial diagnosis**				
Malaria	100 (41.7%)	45 (37.2%)	55 (46.2%)	0.190
Malaria plus bacterial infection	81 (33.7%)	17 (14.0%)	64 (53.8%)	<0.001
Viral infection	40 (16.7%)	40 (33.1%)	0 (0%)	<0.001
Bacterial infection	19 (7.9%)	19 (15.7%)	0 (0%)	<0.001
**Treatment at initial diagnosis**				
Artesunate-Amodiaquine only	106 (44.2%)	45 (37.2%)	61 (51.3%)	0.028
Artesunate-Amodiaquine and Antibiotic	75 (31.2%)	17 (14.0%)	58 (48.7%)	<0.001
Paracetamol only	40 (16.7%)	40 (33.1%)	0 (0.0%)	<0.001
Antibiotic only	19 (7.9%)	19 (15.7%)	0 (0.0%)	<0.001
**Action taken at day 2 review**				
Treatment completed	2 (0.8%)	1 (0.8%)	1 (0.8%)	1.000
Continue medication	192 (80.0%)	117 (96.7%)	75 (63.0%)	<0.001
Admit for treatment	9 (3.7%)	0 (0.0%)	9 (7.6%)	0.002
Change medication	15 (6.2%)	1 (0.8%)	14 (11.8%)	<0.001
Add medication	22 (9.2%)	2 (1.6%)	20 (16.8%)	<0.001
**Action taken at day 7 review**				
Treatment completed	223 (93%)	120 (99.2%)	103 (87.3%)	<0.001
Continue medication	5 (2.1%)	1 (0.8%)	4 (3.4%)	0.211
Admit for treatment	1 (0.4%)	0 (0.0%)	1 (0.8%)	0.496
Change medication	5 (2.1%)	0 (0.0%)	5 (4.2%)	0.029
Add medication	5 (2.1%)	0 (0.0%)	5 (4.2%)	0.029
**Clinical outcome at end of study**				
Completed treatment and well	216 (90.0%)	120 (99.2%)	96 (80.7%)	<0.001
Admitted to hospital	10 (4.2%)	0 (0.0%)	10 (8.4%)	0.001
Remained febrile	13 (5.4%)	1 (0.8%)	12 (10.1%)	0.001
Death	1 (0.4%)	0 (0.0%)	1 (0.8%)	0.496

### Follow up and clinical outcomes

Seven of the 240 patients, three from the intervention group, failed to come for follow up appointments and they were traced to their homes and reviewed. This was to ensure that each patient was clinically safe. At day 2 review, only two (1.6%) of the patients in the intervention group required another laboratory investigation because of persistent fever compared to 13 (10.9%) in the routine care group (p = 0.001). There were significant differences in the clinical outcomes of children in the two groups by day 7. Fever had resolved in 120 (99.2%) of children out of 121 in the intervention group compared to 96 (80.7%) out of 119 in the routine treatment group (p = 0.001). No child in the intervention group required hospitalization whereas in the routine care group 10 (8.4%) children were hospitalized (p = 0.0001). One patient (0.8%) in the intervention group remained febrile at the end of the study compared to 12 (10.1%) in the control group (p = 0.001).

### Accuracy of malaria RDTs in this setting

In the intervention group 60 (49.6%, 95% CI: 40.4% to 58.8%) patients had positive malaria RDTs compared to 49 (40.5%, 95% CI: 31.7% to 41.8%) who had positive blood films by microscopy. Using microscopy as the gold standard, the RDT had a sensitivity of 98.0% (95% CI: 89.1% to 99.9%), a specificity of 83.3% (95% CI: 72.7% to 91.1%), a negative predictive value of 98.4% (95% CI: 91.2% to 100.0%) and a positive predictive value of 80.0% (95%CI: 67.7% to 89.2%).

## Discussion

This study assessed the introduction of a simple test-treat package for management of stable febrile children in an African out-patient clinic in a secondary health care facility. The ‘test’ component of the package consisted of a structured examination of ears, nose and throat, joints and chest, a malaria RDT and urine dipstick testing. The ‘treat’ component was guided by the results of clinical examination and tests, and consisted of a choice of an anti-malarial (artesunate-amodiaquine), an antibiotic (co-amoxiclav) or an antipyretic (paracetamol). These drugs were used singly or in combination according to the diagnosis. The test treat package (intervention) was tested against the routine management (control) of stable febrile illness among children at the OPD by the doctors at SGH. The test-treat package resulted in resolution of fever in >99% of patients except one patient who remained febrile at the end of the study but no child in the test-treat package group required hospital admission.

In contrast, 10.1% of the patients in the routine care had fever at the end of the study, and 8.4% required hospital admission. All patients who had fever at the end of the study were followed up till resolution. Almost all the patients in the routine care group who were prescribed anti-malarials had no laboratory confirmation of malaria infection although blood film microscopy for malaria was available in the hospital. International and local guidelines recommend that malaria should be confirmed if tests are available [19, 22] as this will reduce risk of developing drug resistance and ensure that non-malaria causes of fever are managed appropriately. The significantly higher rates of persistent fever necessitating treatment adjustments or hospital admissions in patients who received routine care compared to those in the intervention group, reinforces the need for stringent implementation of these recommendations to achieve better clinical outcomes.

Despite policies promoting the use of RDTs to investigate fever there is still widespread over-prescribing of anti-malarials in the face of a negative malaria test. One reason may be that health workers often do not have any diagnostic tools for determining the cause of fever and, because they are concerned about missing a possible malaria infection, they default to prescribing anti-malarials. The RDT, which is highly effective in diagnosing malaria as a cause of fever, was included as part of the test-treat package, but the package also incorporated alternative diagnostic and treatment strategies for malaria-negative febrile children. For example, substantial proportions of patients in the intervention group had positive urine dipstick test and were consequently treated with antibiotics and not with anti-malarials. Failure to seek a definitive cause for fever in children puts them at risk of long term complications of the undiagnosed and untreated infections. For example, persistent, untreated urinary tract infections can lead to renal scarring, hypertension and finally chronic renal failure. In addition to the direct cost of prolonged or alternative courses of treatment and hospitalizations, ineffective management of febrile children prolongs ill health and necessitates repeated visits to health facilities, both of which generate an economic and social burden which disproportionately affects families [5, 23].

The rate of anti-malarial use in patients who received routine care was 2.5 times higher than in children who were managed using the test-treat package. Globally, the successful control of malaria is dependent on the continued efficacy of artemisinins. There is already evidence that resistance to artemisinins is appearing in Southeast Asia [24, 25] and the development of resistance is closely linked to overuse of drugs. If the results reflect the situation in other paediatric clinics in malaria endemic areas of sub-Saharan Africa then a half to two thirds of all out-patient prescriptions for anti-malarials in children are unnecessary. This has potentially devastating consequences by promoting the development of drug resistance and jeopardizing efforts to control malaria.

The study had some limitations. The two groups were managed by two different sets of clinicians. This resulted from the study design. The authors went to SGH to test their test treat package against the routine practice of management of fever by the clinician at SGH. It was also conducted during the high malaria transmission season and so the results may not be applicable to areas or seasons with lower transmission rates. However, malaria over diagnosis rates tend to be higher when transmission is lower [23] so our results may underestimate the true benefit of the test-treat package. Possible cross-over effects in patient management was avoided by using separate teams of clinicians to manage the two groups of patients. Both teams of clinicians had the same level of training and clinical experience. The test-treat package is practical and effective for reducing inappropriate prescribing and improving health outcomes in febrile children but it would be important to evaluate it in a variety of other settings including in non-hospital settings such as primary health care clinics. This is a small study of a specific group of patients which was conducted during the high malaria transmission period. The diversity of physicians’ competencies and settings across Africa limit the generalizability of the study. A multi-centre study considering different transmission patterns and with a larger sample size that will enable clustering of patients is needed to confirm the findings.

The potential benefits of such a simple and affordable approach for improving health outcomes in patients, for protecting the economic well-being of their families and for reducing the potential for drug resistance are immense.

### Ethical considerations

Ethical clearance was granted by the Committee on Human Research, Publications and Ethics of the Kwame Nkrumah University of Science and Technology, Kumasi, Ghana.
